# In vitro activity of celastrol in combination with thymol against carbapenem-resistant *Klebsiella pneumoniae* isolates

**DOI:** 10.1038/s41429-022-00566-y

**Published:** 2022-09-27

**Authors:** Mahmoud Saad Abdel-Halim, Momen Askoura, Basem Mansour, Galal Yahya, Amira M. El-Ganiny

**Affiliations:** 1 Microbiology and Immunology Department, Faculty of Pharmacy, Zagazig University, 44519 Zagazig, Egypt; 2 Pharmaceutical Chemistry Department, Faculty of Pharmacy, Delta University for Science and Technology, Gamasa, 11152 Egypt

**Keywords:** Antimicrobials, Infection

## Abstract

*Klebsiella pneumoniae* is an opportunistic pathogen causing nosocomial and community-acquired infections. *Klebsiella* has developed resistance against antimicrobials including the last resort class; carbapenem. Currently, treatment options for carbapenem-resistant-*Klebsiella* (CRK) are very limited. This study aims to restore carbapenem effectiveness against CRK using celastrol and thymol. Clinical *Klebsiella* isolates were identified using biochemical and molecular methods. Antimicrobial susceptibility was determined using disk-diffusion method. Carbapenemase-production was tested phenotypically and genotypically. Celastrol and thymol-MICs were determined and the carbapenemase-inhibitory effect of sub-MICs was investigated. Among 85 clinical *Klebsiella* isolates, 72 were multi-drug-resistant and 43 were meropenem-resistant. Phenotypically, 39 isolates were carbapenemase-producer. Genotypically, *bla*_NDM1_ was detected in 35 isolates, *bla*_VIM_ in 17 isolates, *bla*_OXA_ in 18 isolates, and *bla*_KPC_ was detected only in 6 isolates. Celastrol showed significant inhibitory effect against carbapenemase-hydrolytic activity. Meropenem-MIC did not decrease in presence of celastrol, only 2-fold decrease was observed with thymol, while 4–64 fold decrease was observed when meropenem was combined with both celastrol and thymol. Furthermore, thymol increased CRK cell wall-permeability. Molecular docking revealed that celastrol is superior to thymol for binding to KPC and VIM-carbapenemase. Our study showed that celastrol is a promising inhibitor of multiple carbapenemases. While meropenem-MIC were not affected by celastrol alone and decreased by only 2-folds with thymol, it decreased by 4–64 folds in presence of both celastrol and thymol. Thymol increases the permeability of CRK-envelope to celastrol. The triple combination (meropenem/celastrol/thymol) could be useful for developing more safe and effective analogues to restore the activity of meropenem and other β-lactams.

## Introduction

*Klebsiella* is a Gram-negative bacterium that belongs to the family *Enterobacteriaceae*. *Klebsiella pneumoniae* is the most medically important *Klebsiella species* and one of the major opportunistic pathogens associated with hospital outbreaks [1]. *K*. *pneumoniae* can colonize the intestinal tract, skin, nose and throat of healthy individuals. However, *K*. *pneumoniae* could cause various infections in hospitalized patients, most commonly pneumonia, wound, soft tissue, and urinary tract infections [2]. *K*. *pneumoniae* infections are particularly a problem among neonates, the elderly, and immunocompromised patients. In addition *K*. *pneumoniae* can cause significant number of serious community-acquired infections including pyogenic liver abscess, pneumonia, and meningitis [3].

*Klebsiella pneumoniae* has developed different resistance mechanisms to antimicrobials. These mechanisms include: production of inactivating enzymes, target site modification, reducing the intracellular concentration of antimicrobials either through active expulsion of drug by efflux pump or by changing membrane permeability, or through development of an alternative metabolic pathways [4]. β–lactam is the largest and most important class of antibiotics that have different sub-classes including penicillins, cephalosporins, carbapenems and monobactam. β–lactam antibiotics are widely used in treatment of *K. pneumonia* infections. Bacterial resistance to β–lactams has increased dramatically with the production of β-lactamases [5]. Carbapenems are among the last-line antibiotics that are used against resistant Gram-negative bacteria. The rapid dissemination of carbapenem-resistant *Enterobacteriaceae* (CRE) especially Carbapenem resistant-*Klebsiella* (CRK) represents a global public health threat [6].

Carbapenem-resistance occurs mainly due to production of various types of carbapenemases. Based on the molecular structure and amino acid sequences (ambler classes), carbapenemases belong to class A and D enzymes depend on serine for β-lactams hydrolysis, while class B metallo-betalactamase contains divalent zinc ions important for substrate hydrolysis [7]. In *Enterobacteriaceae*, the most prevalent class A-carbapenemases is the *Klebsiella pneumoniae* carbapenemase (KPC). The class B Metallo-β-lactamases (MBLs) mainly include New Delhi MBL (NDM), Verona Integron-encoded MBL (VIM), and IMP-type carbapenemases. While the most frequently detected class D is the OXA-type β-lactamases [8]. Other carbapenem resistance mechanisms include loss of expression or mutation of porin-encoding genes and overexpression of genes encoding efflux pumps [7].

Carbapenem resistance in Gram-negative pathogens is dramatically limiting treatment options, it is obvious that novel therapies are urgently needed. Recently, new carbapenemase inhibitors with activity against CRE have been approved for clinical use including avibactam, relebactam, and vaborbactam [8]. The development of these inhibitors represents a step in the fight against antimicrobial resistance that needs to be followed by further steps.

Celastrol is a pentacyclic-triterpenoid that is extracted from the root Pulp of the *Tripterygium wilfordii* plant [9]. Celastrol is a component of Chinese medicine that is used to treat cancer, inflammatory, autoimmune, and neurodegenerative diseases [10]. Celastrol has been recently exploited to treat obesity and type 2 diabetes mellitus [11]. In addition, celastrol has been found to exhibit a growth inhibitory activity against Gram-positive bacteria [12]. Furthermore, celastrol was found to reduce staphyloxanthin biosynthesis and biofilm formation in *Staphylococcus aureus* [13, 14]. Besides, celastrol shows antiviral activity against hepatitis viruses [15].

Although celastrol exhibits many biological activities, no previous study has characterized the β-lactamase inhibitory potential of celastrol [16]. However, pentacyclic triterpenoids (e.g., corosolic acid) and polycyclic terpene (e.g., oleanolic acid) were reported as β-lactamase inhibitors. Based on similarities in chemical structure and biological activities [17], celastrol is thought to show a similar activity as a potential β-lactamase inhibitor. This study aims to evaluate the antibacterial activity of celastrol against CRK and to investigate its carbapenemase-inhibitory potential.

## Materials and methods

### Bacterial isolates and chemicals

A total of 85 clinical isolates of *Klebsiella* were recovered from specimens sent to clinical laboratory of Zagazig University hospital, Zagazig, Egypt. Clinical specimens were from different sources including blood, urine, sputum, pus, and endotracheal tube aspirate. Celastrol, Dimethyl sulphoxide (DMSO), Ethylene-diamine tetra-acetic acid (EDTA), Resazurin, and thymol were purchased from Sigma Chemical Co. (St. Louis, MO, USA). Bacterial media and antibiotics disks were purchased from Oxoid (UK).

### Phenotypic and molecular identification of *Klebsiella* species by 16S-rRNA gene sequencing

*Klebsiella* isolates were identified as being Gram-negative rods with lactose fermenting activity on MacConkey agar. Confirmatory tests include: IMViC tests, growth on triple sugar iron and motility tests [18].

Molecular identification was based on sequencing of 16S rRNA gene, gDNA was extracted as described previously [19]. Briefly, a colony of isolate was suspended in 50 µl of nuclease-free water, heated to 100 °C for 10 min using Biometra T-GRADIENT thermocycler (Rudolf-Wissell-Str. Göttingen, Germany). The bacterial debris were removed by centrifugation at 21,000 × *g* for 10 min, the supernatant was used as template in a polymerase chain reaction (PCR). The universal primers 341 F and R806 were used for amplification of bacterial 16S rRNA [20]. These primers were supplied by Sigma Aldrich (Petaluma, USA).

The COSMO PCR RED 2x Master Mix (Willowfort UK) was used. The PCR cycling conditions were as follows: an initial denaturation for 5 min at 94 °C, followed by 30 cycles of denaturation at 94 °C for 1 min, annealing at 55 °C for 1 min, and extension at 72 °C for 2 min, and then a final extension for 5 min at 72 °C. The amplified PCR products were electrophoresed on 2% agarose gel, and photographed with gel documentation system (Cleaver Scientific Ltd, UK). The PCR products were purified using Thermo scientific GeneJET PCR purification kits (Vilnius, Lithuania), according to the manufacturer’s instructions. Purified PCR samples were used for sequencing according to service requirements, where 5 μl of template DNA (20–80 ng) were mixed with 5 μl of 341 F primer (5 pmol μl^−1^). PCR samples were sequenced using the Illumina HiSeq platform using 300 PE chemistry (GATC-Biotech, Konstanz, Germany). The obtained sequences were used to draw the phylogenetic tree and to perform multiple sequence alignment. Phylogenetic analyses were conducted in MEGA11 [21].

### Antimicrobial susceptibility testing

Antimicrobial susceptibility of *Klebsiella* isolates was performed by disk diffusion method according to the clinical and laboratory standard institute (CLSI) guidelines [22]. Briefly, a sterile cotton swab was dipped in Mueller Hinton broth (MHB) with a turbidity equivalent to 0.5 McFarland standards, and used to inoculate the surface of dried Mueller Hinton agar (MHA) plate. The inoculated plates were left to dry for 3–5 min, and the antibiotic disks were placed on them. Plates were incubated at 37 °C for 18 h. The diameter of inhibition zone was measured, recorded, and interpreted according to CLSI (2). The used antibiotic disks include meropenem (MEM, 10 μg), piperacillin-tazobactam (TZP, 100/10 μg), ceftriaxone (CRO, 30 μg), cefepime (FEP, 30 μg), cefoperazone (CFP, 75 μg), aztreonam (ATM, 30 μg), gentamicin (GN, 10 μg), amikacin (AK, 30 μg), azithromycin (AZM, 15 μg), tetracycline (TE, 30 μg), tigecycline (TGC, 15 μg), levofloxacin (LEV, 5 μg), ofloxacin (OFX, 5 μg), trimethoprim-sulfamethoxazole (SXT, 1.25/23.75 μg), and chloramphenicol (C, 30 μg).

### Phenotypic detection of carbapenemase-producing *Klebsiella* by Carba NP test

The Carba NP colorimetric assay for detection of carbapenemase production is based on detection of acidic products due to hydrolysis of imipenem using phenol red indicator [22]. A modified protocol that uses colonies (instead of bacterial extracts) and using 0.1% Triton X-100 as cells lytic agent was used [23]. Briefly, a colony of pure bacterial culture was suspended in tube containing 100 µl of solution A (phenol red 0.05% + ZnSO_4_10 mM + 0.1% (vol/vol) Triton X-100 solution, adjusted to pH= 7.8), or solution B (solution A + 12 mg ml^−1^ imipenem) then vortexed for 10 s and incubated at 35 °C for up to 2 h. The tube containing solution A was used as control and tube containing solution B as test. Carbapenemase-production was indicated by the appearance of orange or yellow color in the test tube while the control tube remained red [23].

### Detection of carbapenemase encoding genes by PCR

Conventional PCR was conducted for the detection of presence of Metallo-β-lactamase genes (*bla*_NDM_ and *bla*_VIM_) or carbapenemases genes (*bla*_OXA-9_ -and *bla*_KPC-1_) in *Klebsiella* isolates. The primers were supplied by IDT (Integrated DNA Technologies, Coralville, Iowa, USA). The sequences of the primers: *bla*_NDM_, *bla*_OXA_, *bla*_KPC-1_ [24], and *bla*_VIM_ [25] are listed in Supplementary Table 1. PCR mixture contained 25 μl of MasterMix, 2 μl of each primer, 2 μl of DNA template, and nuclease free water to 50 μl. The ampilification conditions were: initial denaturation at 95 °C for 3 min followed by 30 cycles of denaturation at 95 °C for 5 s, annealing for 30 s at temperature indicated in supplementary Table 1, and extension at 72 °C for 1 min, followed by final extension at 72 °C for 5 min.

### Determination of minimum inhibitory concentration (MIC) of meropenem, celastrol, and thymol against CRK isolates

Meropenem-MIC was determined by broth microdilution method for CRK isolates that were positive for carbapenemase production. Briefly, 3 colonies were used of each isolate (from overnight culture) to inoculate 5 ml of MHB. Broth cultures were incubated for 18 h at 37 °C. Cultures were then diluted in sterile saline and the turbidity was adjusted to 0.5 McFarland’s standard. Then 1/100 dilution of this suspension was made in sterile MHB. In sterile 96 wells-microplate, serial dilutions of tested chemicals were prepared in sterile MHB including: meropenem (0.5–1024 µg ml^−1^), celastrol (0.5–1024 µg ml^−1^) and thymol (5–2400 µg ml^−1^). 50 µl of the bacterial suspension was added to each well containing the serially diluted chemicals. The plates were incubated for 18 h at 37 °C and examined for bacterial growth. The lowest concentration that inhibited visible bacterial growth was reported as MIC [22]. After determination of meropenem-MIC, isolates with the highest MIC were used in the next analysis, where the MICs of celastrol and thymol were determined by the broth microdilution method.

### Cell viability measurement with Alamar Blue assay

Alamar Blue (Resazurin) assay was performed to assess the effect of sub-MIC of celastrol and thymol on the metabolic activity of CRK as described previously [26]. Briefly, bacterial isolates were incubated alone, with 128 µg ml^−1^ of celastrol, with 300 µg ml^−1^ of thymol, or with a combination of celastrol (128 µg ml^−1^) and thymol (300 µg ml^−1^) for 24 h at 37 °C. Cells were collected by centrifugation at 8000 rpm for 10 min and resuspended in freshly prepared Phosphate-buffered saline (PBS). 0.1 ml of 6.5 mg ml^−1^ resazurin stock solution (prepared in PBS) was added to 0.9 ml of cell suspension. The reaction solutions were incubated in dark for 4 h at 37 °C. Sterile PBS with resazurin was used as blank. The fluorescent intensity of the reduced resazurin (resorufin) was observed at 590 nm emission and 560 nm excitation wavelengths.

### Carbapenemase inhibition assay of crude periplasmic extract

This technique measured the hydrolytic activity of meropenem at a final concentration of (100 μM) using ultraviolet-visible (UV-Vis) spectrophotometry in the presence and absence of enzyme inhibitor. EDTA (reported inhibitor of MBLs) was used to validate the test [27]. Briefly, bacteria were cultured overnight on MHA supplemented with meropenem (4 μg ml^−1^). A standard number of bacterial cells [~5 × 10^8^ cells ml^−1^; optical density (OD) at 600 nm (OD 600) = 0.65] were suspended in 1 ml of 20 mM Tris–HCl buffer containing 2% v/v Triton X. After vigorous mixing, the suspension was left at room temperature for 10 min and then centrifuged at 4000 rpm for 15 min. The supernatant was collected, and 180 µl of the supernatant was added to 96 sterile microtiter-plate. The first well was used to estimate the background absorbance (blank). The second well contained 100 μM of meropenem, the third well contained 100 μM of meropenem and 64 µg ml^−1^ of celastrol, the fourth well contained 100 μM meropenem and 128 µg ml^−1^ of celastrol and the fifth well contained 100 μM meropenem and 300 µg ml^−1^ of thymol. Finally, ddH_2_O was added to each well to make the final volume 200 μl. Hydrolysis of meropenem was measured using a microplate reader (synergy HT BioTek) at 297 nm. Meropenem initial absorbance was measured immediately after the inclusion of the antibiotic. and the reaction plate was incubated at 37 °C for 60 min [28]. The hydrolysis of meropenem was measured after the incubation period. And The hydrolysis index was calculated as follows:

hydrolysis index  =  [Initial Absorbance – Absorbance after 60 min]/Initial Absorbance.

The half maximal inhibitory concentration (IC50) was calculated using Microsoft Excel. IC50 is defined as the minimum concentration of celastrol required for inhibiting the activity of the extracted carbapenemases by 50% Four concentrations of celastrol (0, 32, 64, and 128 µg ml^−1^) were used to determine the percentage of carbapenemases inhibition, then the IC_50_ was calculated.

### Determination of meropenem MIC in presence of sub-MIC of celastrol and/or thymol

The MIC of meropenem was determined in presence of sub-MIC (64 µg ml^−1^ and 128 µg ml^−1^) of celastrol. Furthermore, meropenem MIC was determined in presence of ¼ (300 µg ml^−1^) and 1/8 (150 µg ml^−1^) MIC of thymol. Finally, a triple combination of meropenem, celastrol (64, 128 µg ml^−1^), and thymol (150, 300 µg ml^−1^) was evaluated. The change of meropenem MIC in presence of tested inhibitors was determined using the broth microdilution method as described previously [22].

### Effect of sub-MIC concentrations of thymol on the lytic activities of SDS and Triton X 100 on *K. pneumoniae* outer membrane

This experiment was used to investigate thymol ability to increase the permeability of *Klebsiella* outer membrane to boost the activity of less permeable celastrol [29]. This technique is based on the sensitization of cells to lytic action of the detergents sodium dodecyl sulfate (SDS) and Triton X-100 by thymol. Briefly, a standard inoculum of bacteria (OD_630_ of 0.5) was treated with 300 µg ml^−1^ of thymol for 10 min at room temperature and added to microplate wells that already contained either SDS (0.1 and 1%), Triton X-100 (0.1 and 1%) or buffer solution. Turbidity of the cell suspensions was then monitored with Synergy microplate reader (Agilent, Santa Clara, USA) as described previously [30]. Cell death caused by the sudden influx of these lytic agents was determined by measuring the decrease in OD (Relative turbidity %).

### In silico analysis by molecular docking

The crystal structures of the proteins were retrieved from the Protein Data Bank (https://www.rcsb.org/). These proteins include: the class A carbapenemase KPC-2 (PDB-ID: 3DW0) [31], NDM-1 (PDB-ID: 3SPU) [32], β-lactamase OXA-181 (PDB-ID: 5OE0) [33] and VIM-2 MBL (PDB-ID: 5YD7) [34]. Both celastrol and thymol were drawn into Marvin Sketch of Marvin suite (http://www.chemaxon.com) and lowest energy three-dimensional conformer for each was generated. Dock module of MOE (Molecular Operating Environment) version MOE 2019.0102,2 [35] on a computer having Pentium 1.6 GHz workstation, 512 MB memory using windows operating system, was utilized in docking studies. Tested compounds were docked into the rigid binding pocket of the protein using flexible ligand mode. The free energy of binding of the ligand is estimated using the GBVI/WSA ΔG as a force field-based scoring function [36]. Molecular docking was performed to investigate how ligand binds to protein target [37].

### Statistical analysis

Statistical analysis was performed using GraphPad Prism version 5.0.1 for Windows, GraphPad Software (San Diego, California USA). Carbapenemase inhibition assay and cell viability measurement with Alamar Blue data were analyzed using one-way ANOVA followed by Dunnett’s post hoc test. The Paired t-test was used for analysis of the permeability assay of the outer membrane data. The probability value (*P* < 0.05) was considered as the level of significance.

## Results

### Identification and taxonomic classification of isolated strains

**Fig. 1:**
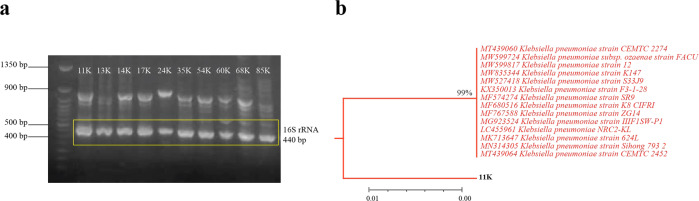
Molecular identification and phylogenetic analysis of bacterial isolates. **a** Gel electrophoresis of PCR products (440 bp) of genes encoding 16S rRNA of suspected *K. pneumoniae*, **b** representative phylogenetic tree of the partial 16S rRNA gene sequence from isolate 11 K compared to sequences of the most related *K. pneumoniae* strains recognized by BLASTN

A total of 85 clinical *Klebsiella* isolates were identified using biochemical tests, isolates with high resistance to carbapenm were confirmed by molecular analysis. Molecular identification was based on 16S rRNA gene sequence, the 16S rRNA gene was amplified by PCR giving a fragment of 440 bp (Fig. 1a). After sequencing, sequences were submitted to the Basic Local Alignment Search Tool of Nucleotides (BLASTN) service in NCBI to align and compare the sequences in relation to reference sequences in the Genebank. BLASTN analysis confirmed that the queried sequences represent a partial sequence of the 16S rRNA of the genus *Klebsiella*. The constructed phylogenetic tree (Fig. 1 b) reveals that all the inspected clinical isolates were completely related to *K. pneumoniae* reference strains.

### Susceptibility of *Klebsiella* isolates to different classes of antimicrobials

Among the 85 clinical *Klebsiella* isolates, 84.70% were multi-drug resistant (MDR) as they were resistant to three or more antimicrobial classes. High percentage of isolates were resistant to ceftriaxone (83.52%), cefoperazone (82.35%), azithromycin (75.3%), tetracycline (72.9%), cefepime (71.76%), trimethoprim /sulfamethoxazole (71.76%), aztreonam (70.85%), piperacillin /tazobactam (64.7%), ofloxacin and levofloxacin (63.5% each). 43 isolates (50.58%) were found to be resistant to meropenem. Less that 50% of isolates were resistant to tigecycline (45.9%) and chloramphenicol (31.8%). The susceptibility profile of *Klebsiella* isolates is shown in Supplementary Fig. 1.

### Phenotypic and genotypic detection of carbapenemase-producing *Klebsiella* isolates

**Fig. 2:**
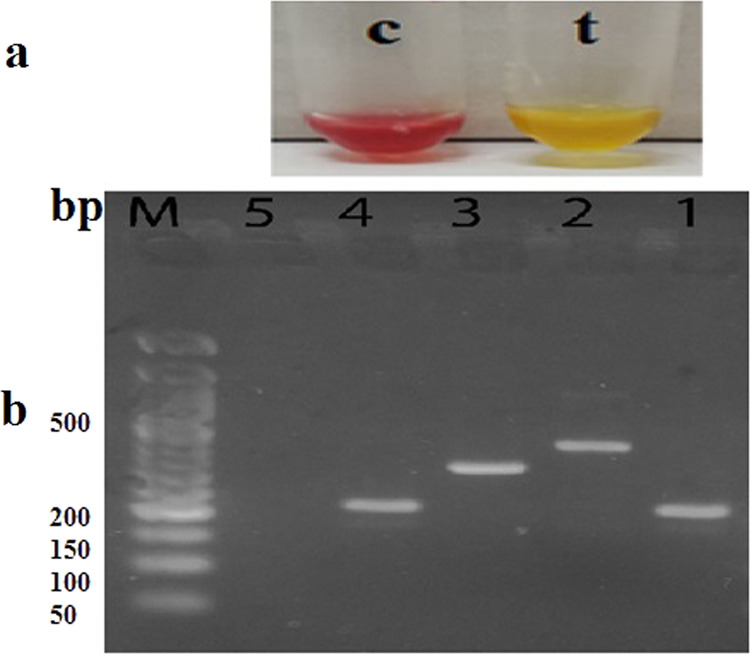
Phenotypic and genotypic detection of carbapenemase activity in *Klebsiella* isolates**. a** The Carba NP test was used for phenotypic analysis, positive results were indicated by the change of color of inoculated tube (t) to yellow: **b** Genotypic detection of carbapenemase genes by PCR in CRK isolates**: lane 1:** *bla*_*NDM-1*_ gene (209 bp), **lane 2:** *bla*_*VIM*_ gene (382 bp), lane 3: *bla*_*OXA*_ gene (315 bp), **lane 4**: *bla*_*KPC*_ gene (209 bp), **lane 5:** negative control**, M**: 50 bp DNA-ladder

Carba NP test was performed on the forty-three isolates that showed resistance to meropenem in susceptibility test. This test reflected that 39 isolates of 43 were carbapenemases producers as shown in Fig. 2a. Furthermore, PCR was performed on the 43 meropenem-resistant isolates to detect genes encoding for carbapenemases. Out of the 43 carbapenem-resistant isolates, the *bla*_NDM-1_ was detected in 35 isolates (81%), the *bla*_*VIM*_ was detected in 17 (39.5%) isolates, the *bla*_*OXA*_ was detected in 18 (41.8%) isolates, and *bla*_*KPC*_ was detected in 6 (13.9%) isolates (Fig. 2b). Only three isolates did not have any of the tested genes.

### Cell viability measurement with Alamar Blue

**Fig. 3:**
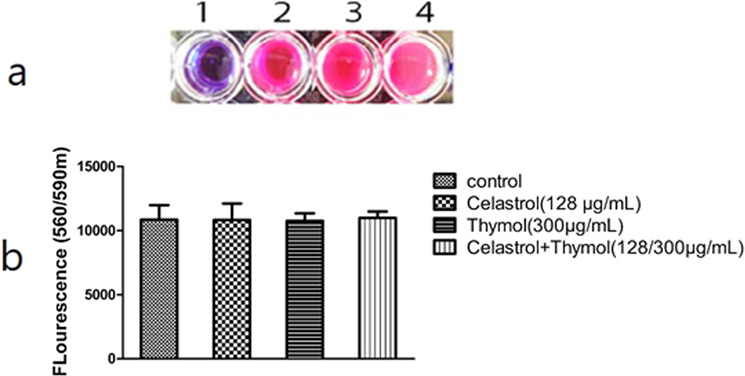
Cell viability measurement with Alamar Blue. **a** Color change of resazurin dye 1: blank, 2: Celastrol, 3: Thymol, 4: Control. **b** fluorescence intensity of the treated culture was not significantly decreased from that of the control culture

This experiment was conducted to determine the effect of celastrol and thymol (alone or in combination) in the used concentrations on the viability of the bacterial cells. As outlined in the materials and methods section, viable cells reduce the non-fluorescing form of this chromogenic indicator dye by cellular dehydrogenases to a pink, fluorescent form, that can be monitored spectrophotometrically. Cell viability is expressed as fluorescence intensity. The experiment revealed that *Klebsiella* isolates cultured in the presence of celastrol (128 µg ml^−1^), thymol (300 µg ml^−1^), or in combination, showed no significant decrease in fluorescence intensity from that of the control culture (Fig. 3).

### Evaluation of the effect of celastrol and thymol on meropenem-MIC in carbapenemase-producing isolates

Eight isolates with the highest MIC were used to study the effect of both celastrol and thymol on meropenem MIC (Table 1). MIC of celastrol for all 8 isolates was higher than 1 mg ml^−1^. MIC of thymol was 1200 µg ml^−1^ for the 8 selected isolates except for isolates 35k and 68k, where the MIC was 600 µg ml^−1^. Table 1 shows the change in MIC of meropenem alone, meropenem in a combination of 128 μg ml^−1^ of celastrol, meropenem in combination with 300 μg ml^−1^ of thymol, and MIC of meropenem in a triple combination.

**Table 1:** The effect of sub-MIC of celastrol, thymol or combination of them on the MIC of meropenem against *K.pneumoniae* isolates

Isolate’s No.	MEM	MEM + Celastrol	MEM + Thymol	MEM + Celastrol + Thymol*
11k	**256**	**256**	**128(2)**	**32(8)**
13k	**256**	**256**	**128(2)**	**8(32)**
14k	**32**	**32**	**16(2)**	**<1(>32)**
17k	**512**	**512**	**256(2)**	**128(4)**
35k	**64**	**64**	**32(2)**	**<1(>64)**
60k	**128**	**128**	**128(0)**	**32(4)**
68k	**256**	**256**	**256(0)**	**64(4)**
85k	**128**	**128**	**128(0)**	**32(4)**

*MEM* meropenem, (fold change in MEM-MIC was listed between brackets)

At sub-MIC of celastrol (128 µg ml^−1^) the eight *Klebsiella* isolates showed no decrease in the MIC of meropenem. At sub-MIC of thymol (150, 300 μg ml^−1^) showed either no change or only 2 fold reduction in MIC of meropenem.The combination of sub- MIC of celastrol (128 μg ml^−1^), and thymol (300 μg ml^−1^) exerted a significant reduction in meropenem MIC (4 to 64 fold reduction).

### Celastrol significantly inhibited the hydrolytic activity of carbapenemases in the crude periplasmic extract

**Fig. 4:**
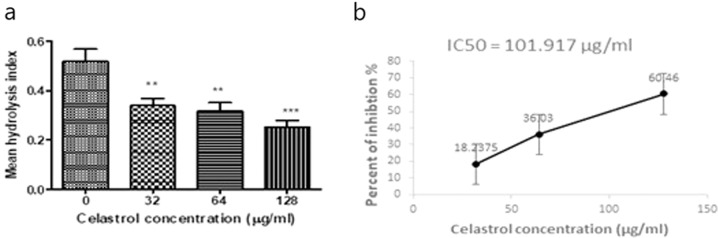
Celastrol inhibitory effect on the hydrolytic activity of the carbapenemases. **a** significant inhibitory effect of different celastrol concentrations on the activities carbapenemases detected by enzyme inhibition assays following co-incubation, **b** Percentage of carbapenemase activities inhibited by celastrol in a concentration-dependent manner. **indicates *P* < 0.01; ***indicates *P* < 0.001

Enzyme inhibition assays were performed to detect the activities of carbapenemases in bacterial culture supernatants when co-incubated with different concentrations of celastrol (32, 64, 128 µg ml^−1^). Celastrol showed a significant inhibitory effect against the activity of carbapenemases when co-incubated in culture supernatants in concentration dependant matter (*P* < 0.05). The celastrol IC_50_ for inhibition of carbapenemases hydrolytic activities was 101.9 μg ml^−1^ (Fig. 4**)**.

### Effect of sub-MIC of thymol on lytic activities of SDS and Triton-X 100 on *K. pneumoniae* outer membrane

The lytic agents (SDS and Triton-X) showed higher lytic activity in presence of sub-MIC (300 µg ml^−1^) of thymol. The lytic activity expressed as relative turbidity percentage of bacterial suspension measured at 630 nm. The result showed that sub-MIC of thymol significantly (*P-*value = 0.0084) increased the permeability of lytic agents through the outer membrane of tested *Klebsiella* isolates (Supplementary Table 2**)**.

### Molecular docking results

**Fig. 5:**
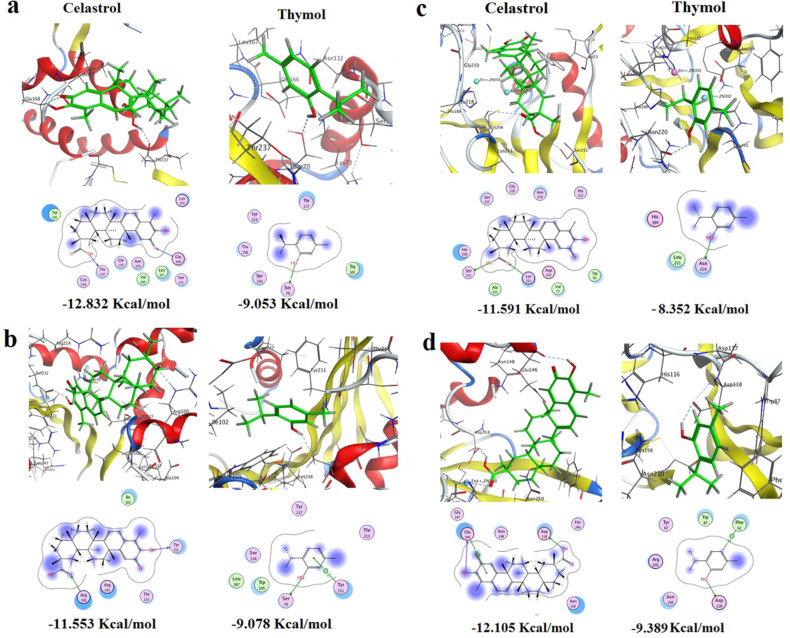
The putative binding modes (2D & 3D) of celastrol and thymol and their free binding energies expressed in Kcal/mol in the active site of the predicted 3D structure of *K. pneumoniae* carbapenemases **a** Class A KPC (KPC-2-3DW0), **b** Class D OXA (OXA-181 5OE0) **c** Class-B NDM (NDM-1 3SPU), **d** Class-B Verona Integron-encoded MBL (VIM-2 5YD7). The blue and cyan shadow of the ligand and active site amino acids respectively indicated strong hydrophobic/hydrophilic interactions

The docking results of celastrol against the crystal structures of the class A carbapenemase KPC revealed that the electronegative oxygen atom doubly bonded to carbon atom of position −11, has constructed an H-bond with the amino acid Glu168. The other terminal of the ligand was firmly stabilized at the core of the pocket *via* another H-bond between the OH of the carboxyl group linked to a carbon atom of position-2 and the conserved H-acceptor amino acid Thr237. Moreover, the hydrophobic/hydrophilic interactions improved binding affinity and total recognition of the ligand inside the core of the pocket ending up with a ligand/receptor complex of −12.8318186 Kcal mol^−1^ stability. While, with thymol, the hydroxyl group of its phenolic structure has formed H-bond with the conserved H-acceptor amino acid Ser70 with a free binding energy of −9.05285263 Kcal/mol (Fig. 5a).

For protein target OXA-181, the H-donor hydroxyl group at position-10 of celastrol formed an H-bond with the conserved amino acid Tyr211, whereas the carboxyl carbonyl (H-acceptor fragment) constructed H-bond with the conserved H-donor amino acid Arg100. Added to hydrophobic/hydrophilic interactions the ligand scored a free binding energy of −11.5534611 Kcal/mol. On the other hand, the phenyl ring of thymol formed an arene-H bond with Tyr211, while its phenolic OH assembled an H-bond with Ser70 at the core of the active site to afford ligand/receptor complex with −9.07757473 Kcal/mol stability **(**Fig. 5b).

On the other hand, docking of celastrol against the New Delhi MBL (NDM) protein target (PDB: 3SPU) showed the role of the carboxyl group at position-2 as its H-acceptor carbonyl and H-donor hydroxyl groups have formed two H-bonds with H-donor Lys211 and H-acceptor Ser251, respectively, with highest free binding energy −11.5906353 Kcal/mol. For thymol, H-bond was constructed between its hydroxyl group and the conserved H-acceptor amino acid Asn220. Besides, the phenyl ring with its hydrocarbon substituents in positions-2, and 5 exhibited conspicuous hydrophobic/hydrophilic interactions giving rise to the highest free binding energy −8.35244465 Kcal mol^−1^ (Fig. 5c).

Furthermore, the docking was extended to VIM-2 Verona Integron-MBL (PDB ID: 5YD7), and the two OH groups of celastrol, at position-10 and of carboxyl group at position-2, displayed two H-bonds with Glu146 and Asp118, respectively. Furthermore, the terminal non-classical phenyl ring formed arene-H-bond with Glu146 leading to the highest free binding energy −12.1051254 Kcal mol^−1^. In addition, the aromatic essential α-amino acid Phe62 established an arene-H-bond with the aromatic hydrogen at position-4 of thymol ligand whose active phenolic OH formed an H-bond with the conserved amino acid Asp118 leading to free binding energy of −9.3893404 Kcal mol^−1^ (Fig. 5d). Overall, celastrol is persistently dominant over thymol in all investigated protein targets and showed the best activity on KPC-2 with a free binding energy of about −12.832 Kcal mol^−1^ followed by VIM-2 with a free binding energy of about −12.105 Kcal mol^−1^.

## Discussion

*Klebsiella pneumoniae* is a major health-care pathogen that is responsible for various infections including urinary tract, pneumonia, meningitis, and pyogenic liver abscess [3]. A total of 85 *Klebsiella* isolates were recovered from various clinical sources, identified by biochemical testing, and confirmed by 16S rRNA sequencing. Antibiotic susceptibility testing showed a higher frequency of MDR (84.70%) than recently reported in other study (50%) [38], the highest resistance rates among tested antibiotics were against cefoperazone and ceftriaxone followed by azithromycin. The lowest resistance rates were against chloramphenicol followed by tigecycline. The low resistance rates to chloramphenicol can be attributed to the limited use of chloramphenicol in clinical practice (topical eye drops) due to its wide range of adverse effects [39]. Tigecycline is a relatively new antibiotic with a low resistance rate [40], in addition, its use is limited to cases of bacteremia and severe pneumonia due to its large volume of distribution resulting in low blood concentration [41].

Approximately 51% of our isolates were CRK which is consistent with another Egyptian study that reported 53.7% resistance to carbapenem in *Klebsiella* [42]. This estimate is slightly lower than the 55% reported from the Europian countries [43] among carbapenemase-producing *Enterobacteriaceae* (EuSCAPE). Higher resistance percentages were reported from Thailand (97%), and United States (63%) [44, 45]. Unfortunately, some local studies reveals that carbapenem resistance rate in Egypt is on the rise [46].

CRK isolates were tested for carbapenemases production and for the presence of four genes representing 3 classes of carbapenemases. Out of the 43 CRK, 40 isolates (93.02%) carried one or more carbapenemase genes. The gene with the highest prevalence was *bla-*_*NDM-1*_ (81%) followed by *bla-*_*OXA*_ (41.8%), and *bla-*_*VIM*_ (39.5%) while *bla-*_*KPC*_ gene has the lowest prevalence (13.9%). Similarly, other studies in Egypt showed high prevalence of *bla-*_*NDM-1*_ *(*80.5 and 94.1%) and reported 36.4 and 47% for *bla*_*VIM*_ [25, 46]. While another study reported lower rate of *bla-*_*NDM-1*_ gene (33.3%), *bla-*_*OXA*_ gene (30.7%), and only 2.5% for *bla-*_*KPC*_ gene [38]. Most of our isolates carry multiple carbapenemase-encoding genes which is consistent with previous report [46]. This can be explained by the fact that genes encoding β-lactamases are mostly carried on mobile genetic elements and thus facilitate their transmition and accumulation within hospital isolates. *Enterobacteriaceae* acquire resistance to carbapenems *via* different mechanisms [47], the most common mechanism is the production of different classes of carbapenemases [8, 46].

CPK-infections are difficult to treat and have limited therapeutic options as seen from our antibiotic susceptibility results. Hence, it is very critical to restore the activity of carbapenems by developing drugs that can be combined with carbapenems to inhibit the action of carbapenemases. Recently two combinations were approved for treatment of carbapenemase-producing bacteria; vaborbactam in combination with meropenem was approved by food and drug administration (FDA) for treatment of complicated urinary tract infections and pneumonia [8]. Vaborbactam only inhibits the class-A carbapenemase KPC with no activity against class-B or D [48]. Relebactam is another FDA-approved carbapenemase inhibitor marketed in combination with imipenem. It does not show in vitro activity against class-D OXA-48 β-lactamases but has potent activity against class-A and class-C β-lactamases [49]. It was reported that triterpenoids such as corosolic acid and polycyclic terpene acids such as oleanolic acid had β-lactamase inhibitory activity [17]. Celastrol has similar chemical structure and biological activity to corosolic and oleanolic acid. Hence, in this study, celastrol was evaluated as potential inhibitor of carbapenemases to restore the activity of meropenem on CRK. Our results showed that celastrol had activity on all tested classes of carbapenemases (KPC, NDM, OXA and VIM-2), while Zhou et al. [17] reported activity against KPC enzyme only. Moreover, the study of Zhou and coworkers used only a standard *E*. *coli* strain to test the activity of corosolic acid, and have reported a maximum MIC of only 14 µg ml^−1^. In our study we tested the effect of celastrol on MDR-clinical CRK isolates (which is more reliable and realistic). It is expected that if corosolic acid is tested on clinical isolates to have much higher MICs.

The antibacterial activity of celastrol was tested against CRK isolates with no inhibitory activity observed for concentrations up to 1024 µg ml^−1^. This is consistent with previous study reported that celastrol showed low MIC against Gram-positive bacteria and no growth inhibitory activity against Gram-negative bacteria in the used concentration [12]. Our study presumed that celastrol possibly acts by the same mechanism on both Gram-positive and Gram-negative bacteria. However, the outer membranes of Gram-negative bacteria act as a permeability barrier, holding celastrol physically distant from its target [50].

Triton-X was used to extract the crude periplasmic enzymes. After incubation with sub-MIC of celastrol (128, 64, and 32 µg ml^−1^), celastrol showed significant inhibition of meropenem hydrolysis in concentration-dependent manner with the highest activity contributed to 128 µg ml^−1^. After this meropenem-MIC was determined in combination with the same concentrations of celastrol. There was no reduction in meropenem-MIC combined with any of the used concentrations. This confirmed the theory that was suggested previously [12] stating that celastrol cannot pass the Gram-negative outer membrane.

The challenge here was to deliver celastrol to the periplasmic space where it can inhibit carbapenemase activity. Disrupting the outer membrane could increase permeability and give celastrol a chance to pass through it. Thymol is a phytochemical that belongs to phenolic monoterpenes [51]. Thymol was described as a natural outer membrane permeabilizer that can increase the intracellular concentration of hydrophobic antibiotics such as erythromycin, azithromycin, sulphamethoxazole/trimethoprim, and novobiocin [29, 51, 52]. One of the intrinsic resistance mechanisms in Gram-negative bacteria is the inner membrane that protects the cell against extracellular toxic compounds. In this membrane, porins regulate the internal accumulation of hydrophilic molecules including antibiotics [53]. Thymol disrupts this membrane by lipophilic action, as it integrates within the polar head groups of the lipid bilayer, inducing alterations of the cell-membrane permeability [54].

To confirm that the sub-MIC of celastrol (128 µg ml^−1^) and thymol (300 µg ml^−1^) do not affect the viability of bacteria, cell viability assay with Alamar-Blue was conducted. It was found that neither celastrol nor thymol or their combination showed any significant decrease in bacteria viability.

Thymol outer membrane disrupting effects were evaluated in this study by testing their effects on the lytic activity of triton X-100 and SDS. The presence of lipopolysaccharide molecules in the outer membrane gives Gram-negative an intrinsic resistance against hydrophilic antibiotics and detergents such as SDS and Triton X-100. It was found that thymol in a concentration 300 µg ml^−1^ (1/4 MIC) sensitized bacterial cells to the cell lytic activity of SDS and Triton X-100 by 15–29% and with a significant difference from untreated culture (*P*-value = 0.0084), indicating a weakening of the outer membrane barrier. Our findings are consistent with that reported previously [29] despite using lower concentration. We used 150 µg ml^−1^ of thymol, but it showed no significant difference.

After establishing the thymol disrupting effect on the protective outer membrane of *Klebsiella* isolates, we evaluated a triple combination of celastrol (128, 64, or 32 µg ml^−1^), and thymol (300 µg ml^−1^) and meropenem. We found that celastrol (128 µg ml^−1^) in presence of thymol reduced meropenem-MIC by (4–64) fold change and rendered two of the test isolates sensitive to meropenem again. This is explained by that thymol increased the permeability of celastrol into the bacterial cell in a concentration high enough to inhibit the hydrolytic activity of carbapenemases thus protecting meropenem and decreasing its MIC. Celastrol at concentrations of 64 or 32 µg ml^−1^ showed no decrease in meropenem-MIC. It was observed that meropenem-MIC was decreased by a maximum of 2-fold when combined with thymol alone.

One of the main issues about celastrol is its toxicity which can strictly limit its clinical applications [55]. Some useful approaches to reduce celastrol toxicity include: combination therapy (which is proposed in our study), synthesis of structural derivatives, development of new formulations and use of more safe routs of administration [56]. For example, it was reported that the use of celastrol-nanoparticles successfully reduced systemic toxicity and also solve the problem of its poor solubility [57]. Furthermore, a recent study suggested a relative safety of celastrol when administered through an oral route [58]. In addition, celastrol can be applied topically for treatment of burn and wound infections caused by CRK. [59]. However the best solution for the toxicity problem is to use celastrol as a lead compound to develop structural analogues with more selectivity to bacterial carbapenemases

The docking results of celastrol and thymol against different carbapenemases revealed the size and bulky structure of celastrol and the distribution of H-donor/ acceptor polar substituents COOH, carbonyl, and OH groups of celastrol were found to be necessary for stabilization of ligand/ receptor complex. While the small size of thymol was found to be less convenient as its binding to protein targets was mainly attributed to its phenolic OH. Of note, the interaction energy expressed in Kcal/mol, reflects how stable is the ligand-protein complex, the lower the interaction energy the more stable the ligand/receptor complex, and the higher the affinity of the compound into the target-receptor.

Since NDM-MβL is one of the most prevalent β-lactamases in *Klebsiella*, a recent in silico study screened natural compounds as potential inhibitors of NDM. The study identified critical residues for NDM inhibition including Ser251, Asn220, Asp124, Lys211, and His122 residues [60]. Another molecular docking study on NDM inhibitors identified six potent inhibitors with free binding energy ranging from −11 to −12 Kcal mol^−1^ and reported that H-bonding was mainly through Lys211 and Asn220 residues [61]. Consistent with these studies, our study showed that celastrol and thymol could bind NDM *via* Lys211, Ser251, and Asn220 amino acid residues.

In addition, a recent study used molecular docking to evaluate the effect of celastrol on some targets for treating thyroid carcinoma [62]. It is worth mentioning that free binding energy scores of celastrol on the selected targets in their study ranged from −7.0 to −9.9 Kcal mol^−1^ [62], which is much lower than the free binding energy scores in our study (ranged from −11.55 to −12.8 Kcal mol^−1^) which means superior activity of celastrol on bacterial carbapenemases compared to human targets.

In conclusion, our investigation revealed that celastrol is a promising inhibitor of carbapenemases. Thymol increases the permeability of the *Klebsiella* envelope to celastrol. Celastrol can be considered as lead compound that could help in the design of new molecules or formulations with more solubility and less toxicity that can be used to restore the activity of carbapenem antibiotics.

## Supplementary information

Supplemental data

## Data Availability

The datasets used /or analyzed in the current study are available from the corresponding author on reasonable request.
