# Supplementary material for: In vitro activity of celastrol in combination with thymol against carbapenem-resistant Klebsiella pneumoniae isolates
Source: J Antibiot (Tokyo). 2022 Sep 27;75(12):679–90. doi: 10.1038/s41429-022-00566-y (PMC9640353; doi:10.1038/s41429-022-00566-y)
Supplement: Supplementary file 1 — Supplemental data [file 41429_2022_566_MOESM1_ESM.docx]

**Supplementary** **Table 1:** Primers used in this study

| **Primer** | **Sequence (5’-3’)** | **Amplicon size (bp)** | **Annealing temp (°C)** |
| --- | --- | --- | --- |
| 341F  R806  *bla*_NDM_ F  *bla*_NDM_ R | CCTACGGGAGGCAGCAG  GGACTACHVGGGTWTCTAAT  GCACACTTCCTATCTCGACATGC  CCATACCGCCCATCTTGTCC | 440  209 | 56  51.5 |
| *bla*_VIM_ F  *bla*_VIM_ R | GATGGTGTTTGGTCGCATA CGAATGCGCAGCACCAG | 382 | 56 |
| *bla*_KPC-1_F  *bla*_KPC-1_R | GTATCGCCGTCTAGTTCTG  CCTTGAATGAGCTGCACAGTG | 209 | 48 |
| *bla*_OXA_ F  *bla*_OXA_ R | CGTCGCTCACCATATCTCCC  CCTCTCGTGCTTTAGACCCG | 315 | 51 |

**Supplementary** **Table 2** Effects of different lytic agents on outer membrane permeability of *Klebsiella* pre-treated with 300 µg/mL of thymol expressed as relative turbidity %. The value of control cells without lytic agents was set at 100%

| Treatment | Turbidity % relative to control | | % of reduction due to thymol |
| --- | --- | --- | --- |
|  | In the absence of Thymol | In the presence of Thymol |  |
| Control | 100% | 100% | 0 |
| SDS 1% | 73% | 55% | 18 |
| SDS 0.1% | 93% | 64% | 29 |
| TRITON-X 1% | 81% | 59% | 22 |
| TRITON-X 0.1% | 86% | 71% | 15 |


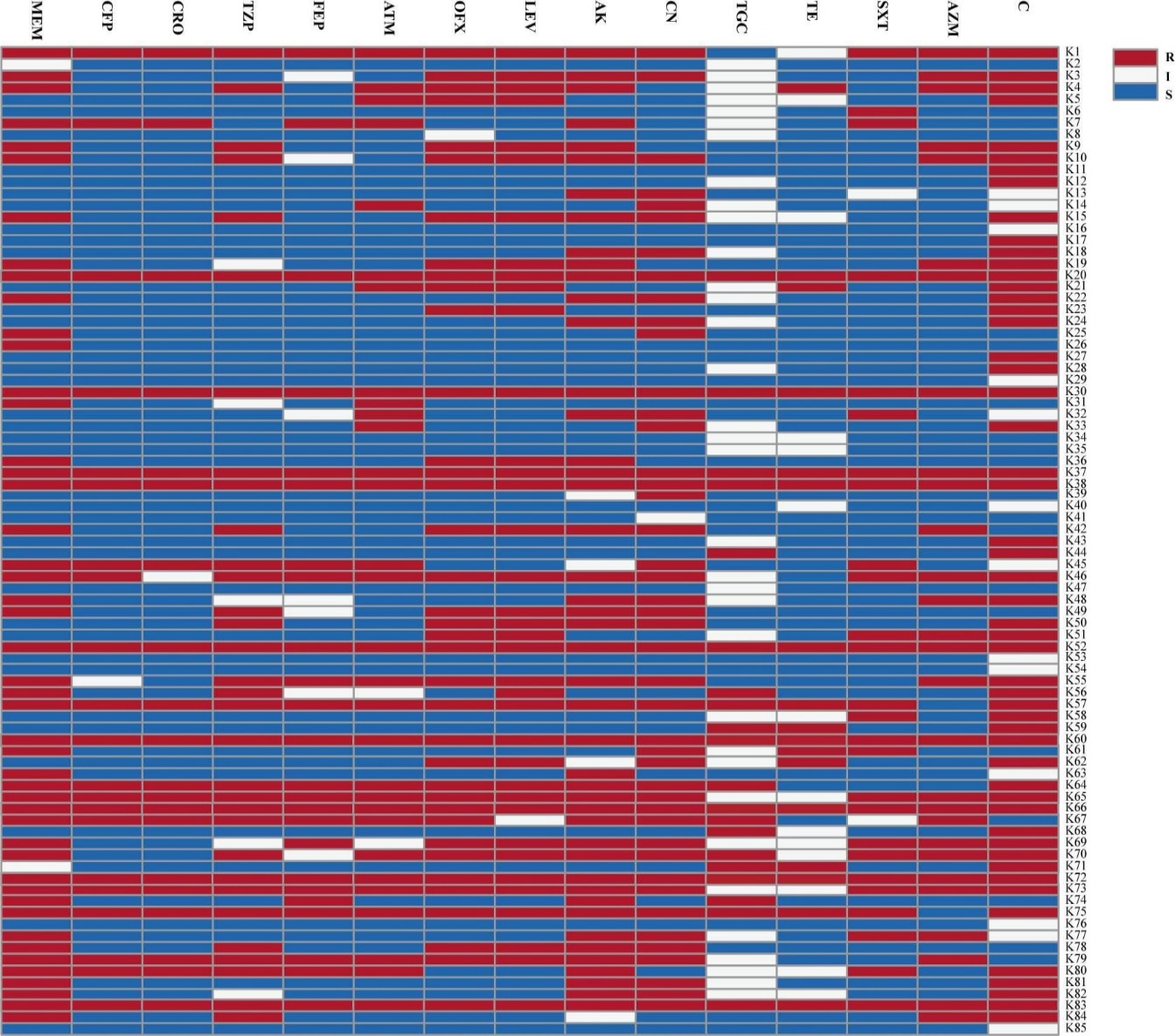


**Supplementary** **Figure 1** **Antimicrobial susceptibility patterns of *Klebsiella species* isolates.** MEM: meropenem, TZP: piperacillin-tazobactam, CRO: ceftriaxone, FEP: cefepime, CFP: cefoperazone, ATM: aztreonam, GN: gentamicin, AK: amikacin, AZM: azithromycin, TE: tetracycline, TGC: tigecycline, LEV: levofloxacin, OFX: ofloxacin, SXT: trimethoprim-sulfamethoxazole, C: chloramphenicol, R: resistant, I: intermediate-resistant, S: sensitive
